# Blood Coagulation-Inspired Fibrin Hydrogel for Portable Detection of Thrombin Based on Personal Glucometer

**DOI:** 10.3390/bios14050250

**Published:** 2024-05-16

**Authors:** Dan-Ni Yang, Shu-Yi Wu, Han-Yu Deng, Hao Zhang, Shan Shi, Shan Geng

**Affiliations:** 1 Chongqing Engineering Research Center of Pharmaceutical Sciences, Chongqing Medical and Pharmaceutical College, Chongqing 401331, China; 2220089@cqmpc.edu.cn (D.-N.Y.); 2120025@cqmpc.edu.cn (H.-Y.D.); 2 Basic Medical College, Chongqing College of Traditional Chinese Medicine, Chongqing 402360, China; wushuyi@cqctcm.edu.cn; 3 The Central Laboratory, The Affiliated Dazu Hospital of Chongqing Medical University, Chongqing 402360, China; shishandazu@163.com

**Keywords:** thrombin activity, personal glucometer, fibrin hydrogel, biosensor, blood coagulation

## Abstract

As one of the biomarkers of coagulation system-related diseases, the detection of thrombin is of practical importance. Thus, this study developed a portable biosensor based on a personal glucometer for rapid detection of thrombin activity. Fibrinogen was used for the detection of thrombin, and the assay principle was inspired by the blood coagulation process, where thrombin hydrolyzes fibrinogen to produce a fibrin hydrogel, and the amount of invertase encapsulated in the fibrin hydrogel fluctuates in accordance with the activity of thrombin in the sample solution. The quantitative assay is conducted by measuring the amount of unencapsulated invertase available to hydrolyze the substrate sucrose, and the signal readout is recorded using a personal glucometer. A linear detection range of 0–0.8 U/mL of thrombin with a limit of detection of 0.04 U/mL was obtained based on the personal glucometer sensing platform. The results of the selectivity and interference experiments showed that the developed personal glucometer sensing platform is highly selective and accurate for thrombin activity. Finally, the reliability of the portable glucometer method for rapid thrombin detection in serum samples was investigated by measuring the recovery rate, which ranged from 92.8% to 107.7%. In summary, the fibrin hydrogel sensing platform proposed in this study offers a portable and versatile means for detecting thrombin using a personal glucometer. This approach not only simplifies the detection process, but also eliminates the need for large instruments and skilled operators, and substantially reduces detection costs.

## 1. Introduction

Thrombin, an enzyme with multifunctional serine protein-hydrolyzing properties, is commonly found in the blood [1,2]. It hydrolyzes soluble fibrinogen into insoluble fibrin, which is involved in the process of blood coagulation [3,4]. In addition, thrombin holds significance in various physiological processes, including inflammatory reactions and vascular tissue repair [5]. Under normal circumstances, thrombin is present in the body as a zymogen. Upon the occurrence of trauma, the zymogen is activated, leading to the production of up to micromolar levels of thrombin to execute hemostatic functions [6]. Elevated levels of thrombin in the body can lead to thrombotic or hemorrhagic disorders, including cardiovascular disease, atherosclerosis, inflammation, and Alzheimer’s disease [7,8,9]. Additionally, thrombin is also used as a therapeutic agent to control bleeding in intraoperative patients. Thus, it is crucial to develop a simple, rapid, cost-effective, and efficient method for the detection of thrombin activity.

Various methods for detecting thrombin activity have been reported, including colorimetric [10,11], fluorescence [12,13], and electrochemical methods [14,15]. These methods are more accurate and have better sensitivity and stability compared to traditional immunoassays. However, the aforementioned thrombin assays have extended analytical times, and necessitate labeled molecules, complex instrumentation, and specialized operators [16], which may restrict their practical application in diverse analytical fields. For example, spectroscopic methods, such as UV and fluorescence, are convenient for real-time monitoring of thrombin activity, but they pose challenges due to the need for specialized instrumentation and trained operators. These methods are limited to compounds with distinct spectroscopic properties and are susceptible to interference from autofluorescence and background absorption. On the other hand, electrochemical methods, known for their high sensitivity, lack automation and require the compounds to be electrochemically active. Therefore, the development of a label-free, economical, and simple method for the detection of thrombin activity is desirable.

Currently, biosensors that offer a rapid response, high sensitivity, and low cost are extensively used in analytical detection. For example, Han et al. [17] designed a paper-based lateral flow sensor for the rapid detection of thrombin based on the conversion of fibrinogen to fibrin, which modulates the viscosity of the solution. After assessing the conditions, only 20.0 µL of sample is required and the detection process can be conducted within 3.0 min, with a limit of detection (LOD) for thrombin at 16.1 mU/mL. Amani et al. [18] designed a colorimetric biosensor for the rapid detection of thrombin using the conversion of fibrinogen to fibrin, which modulates the catalytic ability of fibrinogen-modified gold nanoparticles. After assessing the conditions, the three sensitive colorimetric methods developed for the detxermination of thrombin had a linear range of 20–140 pM and a LOD of 13.41–18.85 pM. However, the reported biosensors still face several challenges, such as difficulty in on-site detection, cumbersome operation, and a lack of portability. These challenges hinder the widespread application of biosensors in various industries. Point-of-care testing (POCT) is a cost-effective, fast responsive, and user-friendly rapid assay technology that has been developed to simplify the detection process and serve patients near the location [19]. Among the various POCT methods, the personal glucometer is the most successful biosensing platform, not only because of its portability, affordability, simplicity of testing, and reliability of results, but also because of its widespread popularity worldwide [20]. To date, the personal glucometer has been widely used for the detection of non-glucose analytes, including the biological thiols [21], alpha-fetoprotein [22], carbendazim [23], cardiac troponin I [24], and enzyme activity [25,26]. Most of the reported specific assays for non-glucose analytes have achieved personal glucometer signal detection through enzyme-catalyzed reactions using labeled or label-free enzymes. However, the main drawbacks of this method are the complexity of the preparation steps, the long response time, and the low analytical sensitivity. To address these limitations, a novel personal glucometer method has been developed for non-glucose analytes. This method involves utilizing chemical cross-linking or entrapping invertase in stimuli-responsive polymers to enable label-free and signal-amplified detection of the analyte. The detection method of the personal glucometer-based stimuli-responsive polymers for non-glucose target detection is as follows: Firstly, enzyme molecules with glucose-producing capability are encapsulated within a stimuli-responsive polymer. Subsequently, the analyte is introduced, which is capable of interacting with the stimuli-responsive polymers, resulting in a change in the structure of the stimuli-responsive polymers and releasing the encapsulated enzyme molecules. Finally, the released enzyme molecules are able to react with the corresponding substrate to produce a signal readout that can be measured using the personal glucometer. For example, Gao et al. [27] developed a DNA tetrahedra-cross-linked hydrogel for rapid detection of DNA adenine methyltransferase based on personal glucometer signal readout. In the presence of DNA adenine methyltransferase, DNA tetrahedra undergoes methylation, leading to hydrogel cleavage and the release of amyloglucosidase. Finally, the released amyloglucosidase catalyzes the production of glucose, which can be measured using the personal glucometer. Based on this principle, the detection limit of 0.001 U/mL for DNA adenine methyltransferase can be achieved. Cao et al. [28] developed an enzyme-encapsulated zeolitic imidazole framework-90 for rapid detection of adenosine-5′-triphosphate combined with personal glucometer signal readout. In the presence of adenosine-5′-triphosphate, the enzyme-encapsulated zeolitic imidazole framework-90 undergoes decomposition and releases the encapsulated enzyme. Finally, the released enzyme catalyzes the production of glucose, which can be measured using the personal glucometer. The LOD value for adenosine-5′-triphosphate was 233 nM.

**Figure 1:**
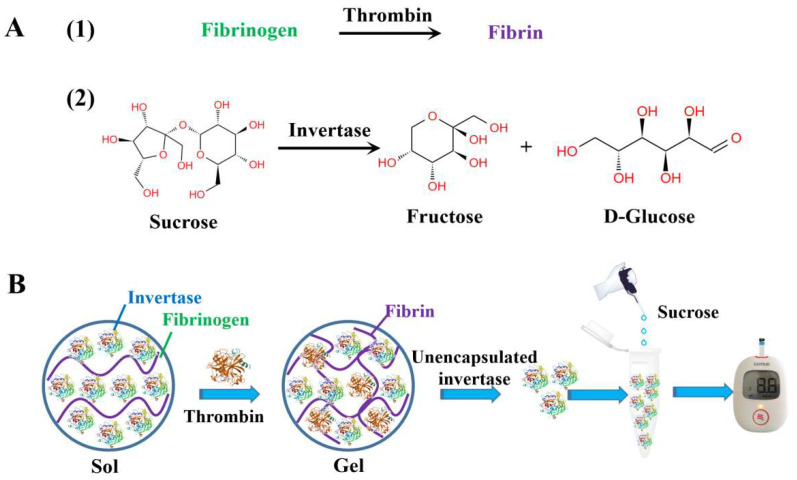
The chemical reactions involved in thrombin detection (**A**), (1) thrombin hydrolyzes fibrinogen to form a fibrin hydrogel, (2) invertase catalyzes sucrose to produce glucose and fructose; and the schematic illustration of the principle of the personal glucometer method for the detection of thrombin activity based on the process of blood coagulation (**B**).

In this study, inspired by the process of blood coagulation, a straightforward and sensitive POCT device has been developed to enable on-site determination of thrombin activity. This was achieved by integrating thrombin-responsive fibrinogen for target recognition and signal transduction with a portable personal glucometer for quantitative signal readout. Fibrinogen not only recognizes thrombin, but also serves as a building block for fibrin hydrogels that encapsulate large amounts of invertase for signal transduction and amplification. In the presence of thrombin, it hydrolyzes fibrinogen to form a fibrin hydrogel that encapsulates a large amount of invertase. The unencapsulated invertase then catalyzes sucrose to produce glucose, which is quantitatively measured using a personal glucometer (Figure 1). The various experimental conditions were systematically examined, including the concentration of fibrinogen, the incubation time between thrombin and fibrinogen, the concentration of invertase, the temperature of the enzymatic reaction, the concentration of sucrose, and the incubation time between invertase and sucrose. Thrombin activity in serum samples was determined using the established glucometer-hydrogel sensor system, demonstrating the potential for the practical application of this method.

## 2. Materials and Methods

### 2.1. Chemicals and Materials

Urea was purchased from Shanghai Macklin Biochemical Technology Co., Ltd. (Shanghai, China). NaCl was purchased from Chongqing Chuandong Chemical Co., Ltd. (Chongqing, China). Sodium bromide and bovine serum albumin were purchased from Titan Scientific Co., Ltd. (Shanghai, China). Sucrose was purchased from Tianjin Kemiou Chemical Reagent Co., Ltd. (Tianjin, China). L-serine hydrochloride, invertase, fibrinogen from bovine plasma, L-glutamic acid monosodium salt, and α-amylase from porcine pancreas were purchased from Yuanye Biological Technology Co., Ltd. (Shanghai, China). (Beijing, China). Thrombin was purchased from Sigma (St Louis, MO, USA). Human serum was purchased from Beijing Solarbio Science and Technology Co., Ltd. (Beijing, China).

### 2.2. Instrumentation

The personal glucometer (Sannuo+) and glucose test strips were purchased from Sinocare Inc. (Changsha, China). A DHG-9035A drying oven and HWS-12 thermostatic water bath were purchased from Shanghai Yiheng Technology Instrument Co., Ltd. (Shanghai, China). The vortex mixer XW-80A type was purchased from Kylin-Bell Lab Instruments Co., Ltd. (Ningbo, China). The SB-4200DT ultrasonic cleaner was purchased from Ningbo Scientz Biotechnology Co., Ltd. (Ningbo, China).

### 2.3. Preparation of Solutions

Fibrinogen (20.0 mg/mL) was prepared by adding it to 10.0 mg/mL NaCl solution. Invertase was prepared by adding it to the above fibrinogen solution. Thrombin solutions with different enzyme activity were prepared by dissolving thrombin in deionized water. The interfering substrates of Cl^−^, Na^+^, urea, α-amylase, L-glutamic acid, and L-serine were prepared using deionized water. Moreover, the 15.0 mg/mL of sucrose was also prepared in deionized water.

### 2.4. Determination of Thrombin Activity

Firstly, 10.0 µL of a fibrinogen solution containing invertase was added to a centrifuge tube, followed by 10.0 µL of thrombin. After incubation at 40 °C for 5.0 min, 10.0 µL of deionized water was added and the mixture was vortexed. Subsequently, 5.0 µL of the solution was pipetted and mixed with 5.0 µL of a sucrose solution, and incubated at 50 °C for 5.0 min. Finally, 1.0 µL of the mixed solution was taken for glucometer analysis. The solution without thrombin was used as a control group and determined according to the following steps: 10.0 µL of deionized water was mixed with 10.0 µL of fibrinogen solution containing invertase and placed in an oven at 40 °C for 5.0 min. Then, 10.0 µL of deionized water was added and after slight shaking, 5.0 µL of supernatant was then incubated with 5.0 µL of sucrose solution in a water bath at 50 °C for 5.0 min. Finally, 1.0 µL of the mixture solution was taken for glucometer analysis. The increment of the glucometer readout (ΔP) was calculated as follows: ΔP = P_blank_ − P_sample_ (where P_blank_ is the glucometer readout of the solution without the thrombin, and P_sample_ is the glucometer readout of the solution with the thrombin).

### 2.5. Selectivity and Interference Study

To confirm the precision and reliability of the established personal glucometer technique for the detection of thrombin activity, the potential influence of various interfering substances in serum samples, such as Cl^−^, Na^+^, urea, α-amylase, L-glutamic acid, and L-serine, was evaluated. To assess selectivity, 10.0 µL of a fibrinogen solution containing invertase was added to a centrifuge tube, followed by 10.0 µL of thrombin or interfering substances. After incubation at 40 °C for 5.0 min, 10.0 µL of deionized water was added and the mixture was vortexed. Subsequently, 5.0 µL of the solution was pipetted and mixed with 5.0 µL of a sucrose solution, and incubated at 50 °C for 5.0 min. Finally, 1.0 µL of the mixed solution was taken for glucometer analysis. The sample without thrombin was used as the control group. The relative PGM readout (%) was calculated as follows: relative PGM readout (%) = P_Interferents_/P_Control_ × 100 (where P_Interferents_ is the glucometer readout of the solution with the interferents or thrombin, and P_Control_ is the glucometer readout of the solution without the thrombin). For the interference study, 10.0 µL of a fibrinogen solution containing invertase was added to a centrifuge tube, followed by 5.0 µL of thrombin and 5.0 µL of interfering substances. After incubation at 40 °C for 5.0 min, 10.0 µL of deionized water was added and the mixture was vortexed. Subsequently, 5.0 µL of the solution was pipetted and mixed with 5.0 µL of a sucrose solution, and incubated at 50 °C for 5.0 min. Finally, 1.0 µL of the mixed solution was then subjected to the glucometer analysis. The sample with thrombin was used as the control group. The relative PGM readout (%) was calculated as follows: relative PGM readout (%) = P_1_/P_0_ × 100 (where P_1_ is the glucometer readout of the solution with the interferents and thrombin, and P_0_ is the glucometer readout of the solution with the thrombin). The final concentrations of thrombin and the interfering substances were 0.005 mg/mL and 0.025 mg/mL, respectively.

## 3. Results and Discussion

### 3.1. Principle of the Glucometer Method for Detecting Thrombin Based on Blood Coagulation

The principle of the glucometer method for detecting thrombin is shown in Figure 1. (1) In a manner similar to blood coagulation, fibrinogen can be enzymatically cleaved to produce fibrin hydrogels with the help of thrombin; (2) The invertase is then introduced into the solution of fibrinogen. Once thrombin is added, the fibrinogen is transformed into a fibrin hydrogel, which effectively immobilizes a portion of the invertase within it; (3) The concentration of free invertase in the solution decreased because the fibrin hydrogel formed and trapped some of the invertase; (4) The hydrolysis of sucrose is catalyzed by invertase to produce glucose and fructose, while the glucometer generates the readout signal by detecting glucose; (5) As the thrombin activity increases, the amount of free invertase in the solution gradually decreases, resulting in a gradual decrease in the amount of glucose produced by hydrolysis. This ultimately leads to a decrease in the signal readout of the glucometer. Therefore, the detection of thrombin activity can be achieved without modifying the glucometer, enzymes, or glucose test strips.

### 3.2. Optimization of Reaction Parameters

**Figure 2:**
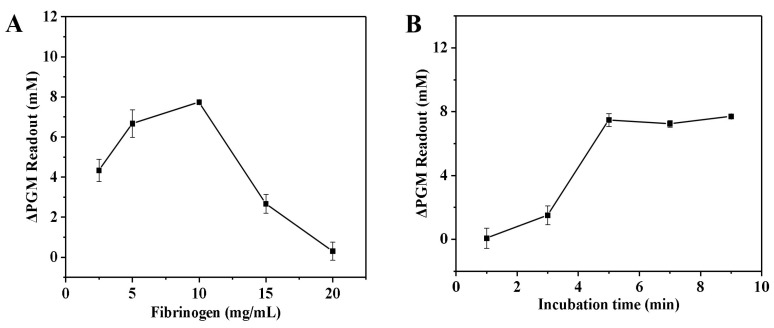
Effect of the fibrinogen concentration (**A**) and the enzymatic reaction time (**B**) on the increment of the glucometer readout. The increment of the glucometer readout was calculated as follows: ΔPGM Readout = P_blank_ − P_sample_ (where P_blank_ is the glucometer readout of the solution without the thrombin, and P_sample_ is the glucometer readout of the solution with the thrombin). Conditions: 0.55 U/mL of thrombin, 0.5 mg/mL of invertase, 5.0 mg/mL of sucrose; thrombin and fibrinogen incubation at 40 °C for 5.0 min, and invertase and sucrose incubation at 50 °C for 5.0 min (**A**); 0.55 U/mL of thrombin, 10.0 mg/mL of fibrinogen, 0.5 mg/mL of invertase, 5.0 mg/mL of sucrose; thrombin and fibrinogen incubation at 40 °C, and invertase and sucrose incubation at 50 °C for 5.0 min (**B**). The error bars represent the standard derivation of three measurements.

The established thrombin assay involves two enzymatic reactions, including the thrombin and invertase systems. For the thrombin system, a neutral environment (deionized water) and mild conditions (40 °C) were chosen for the enzymatic reactions. Moreover, the effects of the final concentration of fibrinogen (2.5–20.0 mg/mL) and the enzymatic reaction time (1.0–9.0 min) on the increment of the glucometer readout were examined. The solution without thrombin was used as a control group. Figure 2A illustrates that as the fibrinogen concentration increased from 2.5 to 10.0 mg/mL, the increment of the glucometer readout gradually increased. However, once the concentration surpassed 10.0 mg/mL, the increment of the glucometer readout gradually decreased. This may be due to the fact that as the concentration of fibrinogen increases, it hinders the proximity of invertase to the substrate sucrose, which leads to a decrease in the rate of enzymatic reaction. As a result, a decrease in the amount of glucose in the solution leads to a decrease in the increment of the glucometer readout. Finally, the fibrinogen concentration of 10.0 mg/mL was chosen for subsequent experiments. As shown in Figure 2B, the increment of the glucometer readout gradually increased with the increase in incubation time and leveled off when the incubation time was greater than 5.0 min. This can be attributed to the fact that as the incubation time increases, more fibrin hydrogel is generated, resulting in a higher amount of encapsulated invertase. As a result, there is a decrease in the amount of invertase present in the solution. When the incubation time exceeded 5.0 min, all of the fibrinogen in the sample was converted to fibrin. At this point, the increment of the glucometer readout no longer increased, even with further increases in incubation time. Therefore, the incubation time between thrombin and fibrinogen was chosen to be 5.0 min for subsequent studies.

**Figure 3:**
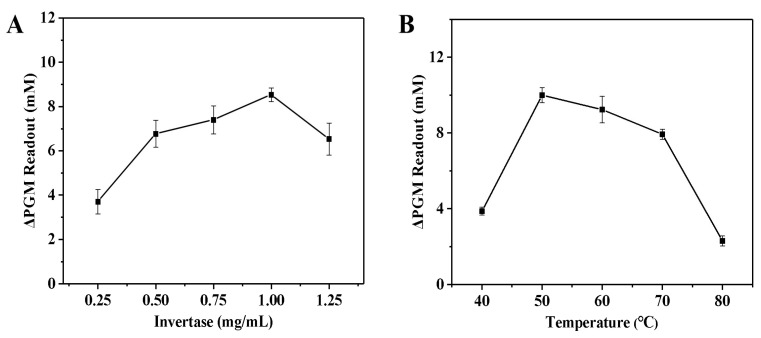
Effect of the concentration of invertase (**A**) and the enzymatic reaction temperature (**B**) on the increment of the glucometer readout. The increment of the glucometer readout was calculated as follows: ΔPGM Readout = P_blank_ − P_sample_ (where P_blank_ is the glucometer readout of the solution without the thrombin, and P_sample_ is the glucometer readout of the solution with the thrombin). Conditions: 0.55 U/mL of thrombin, 10.0 mg/mL of fibrinogen, 5.0 mg/mL of sucrose; thrombin and fibrinogen incubation at 40 °C for 5.0 min, and invertase and sucrose incubation at 50 °C for 5.0 min (**A**); 0.55 U/mL of thrombin, 10.0 mg/mL of fibrinogen, 1.0 mg/mL of invertase, 5.0 mg/mL of sucrose; thrombin and fibrinogen incubation at 40 °C for 5.0 min, and invertase and sucrose incubation for 5.0 min (**B**). The error bars represent the standard derivation of three measurements.

In order to evaluate the invertase systems, the effects of the concentration of invertase (0.25–1.25 mg/mL), enzymatic reaction temperature (40–80 °C), sucrose concentration (2.5–12.5 mg/mL), and the incubation time between invertase and sucrose (3.0–15.0 min) on the increment of the glucometer readout were examined. As shown in Figure 3A, the increment of the glucometer readout gradually increased as the invertase concentration increased, and gradually decreased when the concentration was greater than 1.0 mg/mL. This may be due to the fact that as the amount of invertase increases, the amount of invertase encapsulated in the fibrin hydrogel also increases. However, when the amount of invertase exceeds the enzyme loading of the fibrin hydrogel, even if the amount of invertase is further increased, the amount of invertase encapsulated in the fibrin hydrogel no longer increases. This leads to an increase in the amount of free invertase remaining in solution. On the other hand, the increment of the glucometer readout (ΔP) was calculated as follows: ΔP = P_blank_ − P_sample_ (where P_blank_ is the glucometer readout of the solution without the thrombin, and P_sample_ is the glucometer readout of the solution with the thrombin). Thus, as the amount of invertase increases, the P_blank_ value also increases, but the P_sample_ value increases more significantly. Accordingly, the ΔP value tends to decrease. Finally, the invertase concentration of 1.0 mg/mL was used for the next study. Next, the influence of the enzymatic reaction temperature on the increment of the glucometer readout was analyzed. In Figure 3B, the increment of the glucometer readout rises rapidly with increasing temperature, but decreases slowly when the temperature exceeds 50 °C. This may be attributed to the fact that the inactivation of the enzyme at high temperatures. Finally, the optimum temperature of 50 °C was chosen for subsequent optimization of conditions.

**Figure 4:**
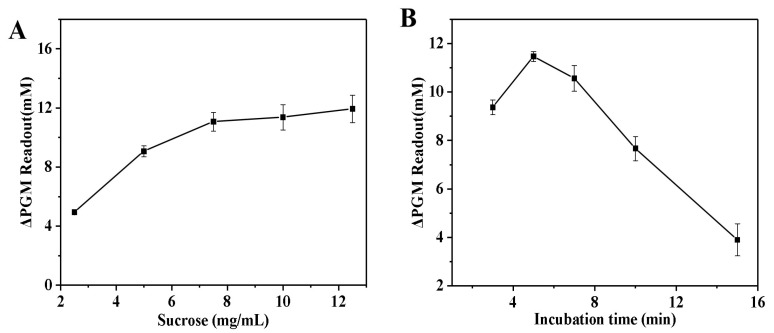
Effect of sucrose concentration (**A**) and the enzymatic reaction time (**B**) on the increment of the glucometer readout. The increment of the glucometer readout was calculated as follows: ΔPGM Readout = P_blank_ − P_sample_ (where P_blank_ is the glucometer readout of the solution without the thrombin, and P_sample_ is the glucometer readout of the solution with the thrombin). Conditions: 0.55 U/mL of thrombin, 10.0 mg/mL of fibrinogen, 1.0 mg/mL of invertase; thrombin and fibrinogen incubation at 40 °C for 5.0 min, and invertase and sucrose incubation at 50 °C for 5.0 min (**A**); 0.55 U/mL of thrombin, 10.0 mg/mL of fibrinogen, 1.0 mg/mL of invertase, 7.5 mg/mL of sucrose; thrombin and fibrinogen incubation at 40 °C for 5.0 min, and invertase and sucrose incubation at 50 °C (**B**). The error bars represent the standard derivation of three measurements.

Figure 4A illustrates the relationship between sucrose concentration and the increment of the glucometer readout. The increment of the glucometer readout showed a steady increase as the sucrose concentration increased, reaching a plateau when the concentration exceeded 7.5 mg/mL. This can be attributed to the fact that at lower substrate concentrations, the increment of the glucometer readout increases with increasing substrate concentration. As the substrate concentration continues to increase, the increment of the glucometer readout gradually slows down. Continuing to increase the substrate concentration, the increment of the glucometer readout no longer increases, at which point all the active centers of the enzyme have been saturated with substrate. Thus, the sucrose concentration of 7.5 mg/mL was used for the next study. Finally, the incubation time between invertase and sucrose was investigated. As shown in Figure 4B, the increment of the glucometer readout steadily increased with increasing incubation time. After 5.0 min had passed, there was a gradual decrease in the increment of the glucometer readout. The optimized conditions for the invertase systems were as follows: invertase concentration of 1.0 mg/mL; the optimum temperature of 50 °C; the sucrose concentration of 7.5 mg/mL; incubation time of 5.0 min.

### 3.3. Analytical Performance

**Figure 5:**
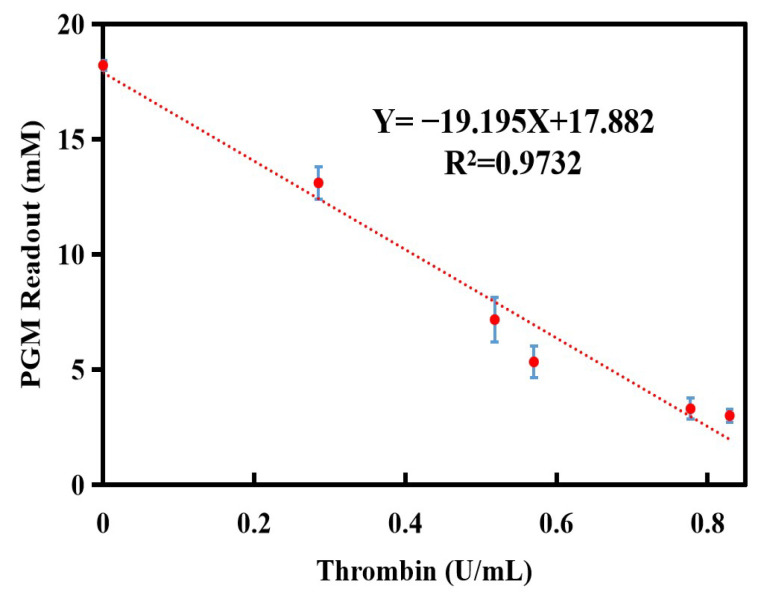
Calibration curve for the detection of thrombin activity using the personal glucometer method. Conditions: 10.0 mg/mL of fibrinogen, 1.0 mg/mL of invertase, 7.5 mg/mL of sucrose; thrombin and fibrinogen incubation at 40 °C for 5.0 min, and invertase and sucrose incubation at 50 °C for 5.0 min. The error bars represent the standard derivation of three measurements.

To assess the repeatability of the method in detecting thrombin, the relative standard deviation (RSD%) of the glucometer readout was determined. After five consecutive measurements, the RSD% value of the glucometer readout was determined to be 7.0%. The results showed that the established thrombin assay had good reproducibility and accuracy. Subsequently, a linear equation was constructed with thrombin activity serving as the x-axis and the glucometer readout as the y-axis. As shown in Figure 5, there is a good linear relationship between the glucometer readout and thrombin activity, as indicated by the following equation: the glucometer readout (mM) = −19.195 × C_Thrombin_ (U/mL) + 17.882 (R^2^ = 0.9732). The linear range of the thrombin assay was ranged from 0 to 0.8 U/mL and the LOD value was calculated as 0.04 U/mL (LOD = 3∗δ/S, where S is the slope of the calibration curve and δ is the standard deviation of eleven blank assays). Compared to the nanomaterial labels method [29] and lateral flow assay [17] used for the detection of thrombin activity, this method has a wider linear range (0–0.8 U/mL) than the nanomaterial labels method (4–128 U/L) and lateral flow assay (0–85.6 mU/mL) mentioned above, and its sensitivity is comparable to that of the two methods. Additionally, this method avoids the use of large instruments, labeling treatments of biomolecules, and specialized personnel, and only requires fibrinogen, enzyme reagents, and glucose test strips for the entire testing process.

### 3.4. Selectivity and Interference Study

**Figure 6:**
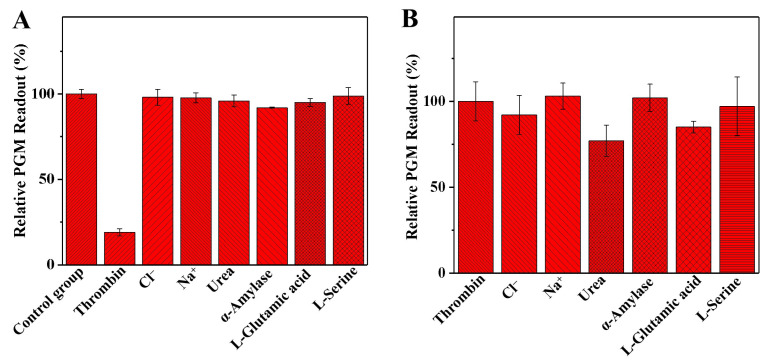
The selectivity (**A**) of the developed personal glucometer method for the detection of thrombin activity, the relative PGM readout (%), was calculated as follows: relative PGM readout (%) = P_Interferents_/P_Control_ × 100 (where P_Interferents_ is the glucometer readout of the solution with the interferents or thrombin, and P_Control_ is the glucometer readout of the solution without the thrombin). The interference study (**B**) of the developed personal glucometer method for the detection of thrombin activity, the relative PGM readout (%), was calculated as follows: relative PGM readout (%) = P_1_/P_0_ × 100 (where P_1_ is the glucometer readout of the solution with the interferents and thrombin, and P_0_ is the glucometer readout of the solution with the thrombin). Conditions: 0.005 mg/mL of thrombin, 10.0 mg/mL of fibrinogen, 1.0 mg/mL of invertase, 7.5 mg/mL of sucrose, 0.025 mg/mL of interfering substances; thrombin and fibrinogen incubation at 40 °C for 5.0 min, and invertase and sucrose incubation at 50 °C for 5.0 min. The error bars represent the standard derivation of three measurements.

To assess the selectivity of the developed method in detecting thrombin, the potential interferents in serum samples, such as Cl^−^, Na^+^, urea, α-amylase, L-glutamic acid, and L-serine, were determined. The solution without a thrombin sample was chosen as a control group. As shown in Figure 6A, the addition of thrombin had the greatest effect on the relative glucometer readout, while the addition of other interferents had a negligible impact on the relative glucometer readout. This indicates that the method used in this study has good selectivity for thrombin detection. On the other hand, the interference study was also evaluated. As shown in Figure 6B, the relative glucometer readout was only slightly affected by urea and L-glutamic acid. The other interferents had a negligible effect on the relative glucometer readout, indicating that the analytical method established in this study has good accuracy for thrombin activity detection.

### 3.5. Real Sample Analysis

As a proof of concept, a serum sample spiking recovery test was performed to evaluate the reliability of the portable glucometer method for rapid thrombin detection. Serum samples were diluted 1000-fold before undergoing subsequent analysis experiments. As shown in Table 1, the recoveries ranged from 92.8% to 107.7% when thrombin was added to serum samples at three different activities (0.26, 0.52, and 0.77 U/mL). These results indicate that the developed rapid and simple glucometer assay is capable of detecting thrombin in real samples.

## 4. Conclusions

In this work, inspired by blood coagulation, a detection platform for thrombin activity is proposed. Firstly, fibrin hydrogel was successfully prepared as an enzyme immobilization carrier for efficient detection of thrombin activity using a personal glucometer. The fibrin hydrogel was prepared by hydrolyzing fibrinogen using thrombin. The method for preparing the fibrin hydrogel is simple and feasible, making it a promising approach for developing new functional materials. The method employed in this study offers the advantage of extending the personal glucometer assay to rapidly detect thrombin activity, similar to the detection of glucose in blood samples. In addition, the selectivity and interference study indicates that the method used in this study has good selectivity and accuracy for thrombin detection. The developed method exhibits an analytical performance of up to 0.04 U/mL for thrombin. In conclusion, this study presents an economical, efficient, and sensitive method for detecting thrombin activity using the fibrin hydrogel-embedded enzyme signal amplification method.

## Figures and Tables

**Table 1:** Recovery study of thrombin in serum sample (*n =* 3).

Sample	Added (U/mL)	Found ± SD (U/mL)	Recovery (%)
Human serum	0	0	-
	0.26	0.24 ± 0.03	92.8
	0.52	0.56 ± 0.05	107.7
	0.77	0.76 ± 0.02	97.5

## Data Availability

The raw data supporting the conclusions of this article will be made available by the authors upon request.
